# Molluscum contagiosum virus protein MC089 inhibits interferon regulatory factor 3 activation

**DOI:** 10.1099/jgv.0.002015

**Published:** 2024-08-21

**Authors:** Mariya Al Hamrashdi, Carla Sanchez Perez, Darya A. Haas, Jyoti Vishwakarma, Andreas Pichlmair, Andrew G. Bowie, Gareth Brady

**Affiliations:** 1 Trinity Health Kidney Centre, Trinity Translational Medicine Institute, Trinity College Dublin, St. James’ Hospital Campus, Dublin, Ireland; 2 Technical University of Munich, School of Medicine, Institute of Virology, Munich, Germany; 3 German Centre for Infection Research (DZIF), Munich Partner Site, Munich, Germany; 4 School of Biochemistry and Immunology, Trinity Biomedical Sciences Institute, Trinity College Dublin, Dublin, Ireland

**Keywords:** IKKε, innate immunity, MAVS, MC089, molluscum contagiosum virus, NAP1, TBKBP1

## Abstract

Molluscum contagiosum virus (MCV) is a human-specific poxvirus that causes a highly common but mild infection characterized by distinctive and persistent papular skin lesions. These lesions can persist for long periods without an effective clearance response from the host. MCV, like all poxviruses, encodes multiple known immunosuppressive proteins which target innate immune signalling pathways involved in viral nucleic acid sensing, interferon production and inflammation which should trigger antiviral immunity leading to clearance. Two major families of transcription factors responsible for driving the immune response to viruses are the NF-κB and the interferon regulatory factor (IRF) families. While NF-κB broadly drives pro-inflammatory gene expression and IRFs chiefly drive interferon induction, both collaborate in transactivating many of the same genes in a concerted immune response to viral infection. Here, we report that the MCV protein MC089 specifically inhibits IRF activation from both DNA- and RNA-sensing pathways, making it the first characterized MCV inhibitor to selectively target IRF activation to date. MC089 interacts with proteins required for IRF activation, namely IKKε, TBKBP1 and NAP1. Additionally, MC089 targets RNA sensing by associating with the RNA-sensing adaptor protein mitochondrial antiviral-signalling protein on mitochondria. MC089 displays specificity in its inhibition of IRF3 activation by suppressing immunostimulatory nucleic acid-induced serine 396 phosphorylation without affecting the phosphorylation of serine 386. The selective interaction of MC089 with IRF-regulatory proteins and site-specific inhibition of IRF3 phosphorylation may offer a tool to provide novel insights into the biology of IRF3 regulation.

## Introduction

Molluscum contagiosum virus (MCV) is a human-adapted poxvirus with a linear double-stranded DNA genome that primarily infects keratinocytes, the principal cell type of the epidermis, causing the formation of distinct skin lesions with typically minimal inflammation [1,2]. The infection does not usually require stringent treatment as it only appears severe in immunocompromized patients suggesting a role of adaptive immunity in its restriction [3,4]. Although keratinocytes drive efficient antiviral immunity, possessing a plethora of pattern recognition receptors (PRRs) [5], MCV infection can last from months to years likely due to effective evasion and inhibition of human antiviral sensing, inflammation and interferon pathways.

After sensing of virus infection occurs, two major families of transcription factors collaborate to drive primary innate immune gene expression: NF-κB and interferon regulatory factors (IRFs). The NF-κB family induces both pro-inflammatory cytokine and type I interferon (TI-IFN) genes with more rate-limiting control over the former [6,8]. The IRF family is more directly involved in driving the expression of TI-IFN genes [9,12]. However, both families collaborate on regulating overlapping genes in the antiviral response [6]. The activation of both sets of transcription factors is controlled by upstream signalling cascades through phosphorylation and ubiquitination of a series of adaptor proteins and kinases after sensing viral pathogen-associated molecular patterns by PRRs [13].

IRF3 plays the strongest role in TI-IFN gene regulation in the initial phase of antiviral innate immunity in most cells [14]. The fact that viruses routinely evolve inhibitors of IRF3 activation highlights its importance in early antiviral defences [15,22]. IRF3 is strongly activated by multiple pathways that sense viruses like the cytosolic DNA sensor cyclic GMP–AMP synthase (cGAS) and the RNA sensors toll-like receptor 3 (TLR3) and retinoic acid-inducible gene I (RIG-I)-like receptors (RLRs), which trigger downstream signalling cascades through stimulator of interferon genes (STING), TIR-domain-containing adapter-inducing IFNβ (TRIF) and mitochondrial antiviral-signalling protein (MAVS), respectively [23,25]. Subsequently, they activate complexes containing the adaptors TANK-binding kinase 1-binding protein 1 (TBKBP1), also known as similar to NAP1 TBK1 adaptor (SINTBAD), TRAF family member-associated NF-kappa-B activator (TANK) and NF-kappa-B-activating kinase-associated protein 1 (NAP1), which regulate the kinases IκB kinase epsilon (IKKε) and TANK-binding kinase-1 (TBK1). Once activated, TBK1 and IKKε phosphorylate IRF3 in its C-terminal domain, resulting in its dimerization and nuclear translocation to bind to accessible interferon-stimulated response element (ISRE)–containing gene promoters, most notably in TI-IFN genes [24,26,30].

While the NF-κB-activating IKK complex [consisting of two kinases IKKα and β with a regulatory protein (IKKγ/NEMO)] is relatively well characterized at this point, many questions remain about the equivalent activation complexes which regulate IRF3 [31]. Specifically, while the NF-κB-regulating complex has one regulatory protein IKKγ/NEMO, the IRF3-activation complex has three, TBKBP1, NAP1 and TANK, which compete for interaction with IRF-activating kinases. Additionally, the precise distinguishing roles of TBK1 and IKKε in IRF3 activation are still unclear [32,37]. Also not fully clear is the importance of IRF3 phosphorylation sites in each stage of its activation. Inactive IRF3, which is in the cytoplasm of most cells, is exposed to both phosphorylation and dimerization upon virus infection that allow for its nuclear translocation and subsequent transactivation of TI-IFN genes [38,39]. Phosphorylation of IRF3 serine residues 386 and 396 was shown to play different roles in dimerization, nuclear translocation and transactivation, but the exact roles of each site are still debatable [39,42].

Here, we report the discovery of MC089 as a novel, specific inhibitor of IRF3 activation from both DNA- and RNA-sensing pathways which targets IKKε, TBKBP1, NAP1 and MAVS in association with mitochondria. Interestingly, MC089 prevents the phosphorylation of IRF3 on serine 396 without affecting the phosphorylation of serine 386. MC089 specificity of IRF3-regulatory component targeting and IRF3 site phosphorylation inhibition suggests that this MCV inhibitor may serve as a useful tool to investigate key aspects of IRF3 biology.

## Methods

### Cell culture

HEK293T cells were maintained in Dulbecco’s Modified Eagle Medium (DMEM) (1 ×) (Gibco # 61965026) supported with 10% (vol/vol) FBS (Sigma-Aldrich # F9665) at 37 °C and 5% CO_2_. The antibiotic used was periodically alternated between 1% (vol/vol) penicillin–streptomycin (Gibco # 15140122) and 0.1% (vol/vol) gentamicin (Sigma-Aldrich # G1397) to maintain sterility. For passaging, cells were trypsinized using 0.05% trypsin-EDTA (Gibco # 25300054), following a double washing step with sterile PBS (Gibco # 10010056).

### Plasmids and oligonucleotides

MCV subtype 1 MC089L was custom synthesized by GenScript and subcloned into KpnI and NotI sites of the pCEP4 expression vector (Invitrogen # V04450) tagged with a C-terminal FLAG (DYKDDDDK) or an HA (YPYDVPDYA) epitope. Similarly, MCV subtype 1 MC020L was cloned into the pCEP4 vector tagging a C-terminal FLAG (DYKDDDDK). For CdCl_2_-conditional expression, MC089L was subcloned into KpnI and NotI restriction sites of the pMEP4 vector (Invitrogen) and fused with a C-terminal FLAG tag. Other plasmids were acquired as referred to previously [43,45]. MC020 was subcloned from previously described pCEP4-MC020-FLAG into the KpnI and HindIII sites of a pCEP4-HA construct, resulting in a C-terminal HA tag. The vaccinia virus protein VVC6 was constructed as described previously [45].

### Antibodies

Primary antibodies used for immunoblotting were anti-FLAG M2 (Sigma-Aldrich # F3165), anti-HA (Biolegend # 901515) and anti-β-Actin (Sigma-Aldrich # A5316). Goat anti-mouse (IRDye 680RD) (LI-COR # 92668070), goat anti-rabbit (IRDye 680RD) (LI-COR # 92668071) and goat anti-rabbit (IRDye 800CW) (LI-COR # 92632211) were used as secondary antibodies. Phosphospecific antibodies were obtained from the RIG-I pathway sampler kit (Cell Signaling Technology # 8348), except for anti-IRF3 (phospho S386) (Abcam # 76493). Other antibodies used include an anti-MAVS antibody (Cell Signaling Technology # 3993) and an anti-TBKBP1 antibody (Sigma-Aldrich # ZRB1932). For confocal microscopy, Alexa Fluor 488 FLAG tag antibody (Invitrogen # MA1142A488) and Alexa Fluor 647 HA tag antibody (Invitrogen # 26183A647) were used.

### Reporter gene assays

HEK293T cells were seeded at 2×10^5^ cells ml^−1^ in 96-well culture plates. The cells were transfected 24 h later with 80 ng/well of the indicated firefly luciferase reporter gene (κB-luciferase, ISRE-luciferase and IFNβ promoter luciferase), 40 ng/well of pGL3-*Renilla* control and the indicated amounts of signalling element plasmid constructs and MCV ORFs. The final volume of plasmids added was adjusted to 220 ng/well using the empty vector control (pCMV-HA). The next day, cells were infected with Sendai virus (SeV) (ECACC) for 24 h, transfected with 1 µg ml^−1^ of Poly(dA:dT) for 16 h or directly lysed using passive lysis buffer (Promega technical bulletin # TB281). GeneJuice reagent (Merck # 70967) was used to transfect the cells with plasmids, while Lipofectamine 2000 reagent (Invitrogen # 11668019) was used for Poly(dA:dT) transfection. Supernatants were aspirated, and cells were incubated with 50 µl/well of passive lysis buffer for 15 min on the shaker. Using white luminometer plates, 20 µl/well of lysates was mixed with 40 µl/well of luciferase assay mixture (LAM 1 ×) or 40 µl/ well of coelenterazine (0.1% (vol/vol)) (Biotium # 10110). LAM was prepared using 20 mM tricine, 2.67 mM magnesium sulphate heptahydrate, 0.1 mM EDTA, 33.3 mM dithiothreitol (DTT), 530 µM ATP disodium salt, 270 µM acetyl coenzyme A sodium salt, 420 nM d-luciferin, 5 µM sodium hydroxide and 9.7 mM magnesium carbonate hydroxide pentahydrate. LAM (d-luciferin) measured firefly luciferase activity, while coelenterazine measured *Renilla* luciferase activity through light emission. For the Ras-dependent Elk1-Gal4 reporter assay and the IRF3-GAL4 assay, 80 ng of the pFR luciferase plasmid containing Gal4-binding sites, 5 ng of the pFA-Elk1-GAL4 expression vector or 5 ng of the IRF3-GAL4 expression vector and 50 ng of HA-tagged RasVHa or 10 ng TRIF were used. Luciferase activity was read using a Luminoskan microplate luminometer (Thermo Scientific). Firefly luciferase values were then normalized with associated *Renilla* luciferase values for each well to account for transfection efficiency before further processing of data.

### Immunoblotting

HEK293T cells were seeded at 5×10^5^ cells/well in six-well plates and transfected 24 h later with a total of 3 µg of plasmid via GeneJuice. The next day, supernatants were aspirated, and the cells were lysed with 180 µl/well of sample lysis buffer [1 M Tris-HCl (pH 6.8), 2% (wt/vol) sodium dodecyl sulphate, 10% (vol/vol) glycerol, 0.1% (wt/vol) bromophenol blue, 100 mM DTT, 1 µl ml^−1^ benzonase and dH_2_O] for 5 min on ice. Lysates were then boiled for another 5 min. Lysates (20 µl/well) were resolved by 10–15% SDS-PAGE, and Western blotting was performed using nitrocellulose membranes (VWR # 10600012). Membranes were blocked to reduce non-specific binding for 1 h using 3% (wt/vol) BSA dissolved with PBS (1 ×) and 0.05% (vol/vol) Tween 20, except for phosphorylation antibodies as BSA was dissolved with TBS and Tween 20 to minimize potential effects on protein phosphorylation from PBS. Probing with primary antibodies was performed according to the manufacturer’s specifications using blocking buffer for dilution: anti-FLAG (1/5000), anti-HA (1/1000) and anti-β-actin (1/10,000). Following overnight incubation at 4 °C, membranes were washed with PBS (1 ×) and 0.05% (vol/vol) Tween 20, probed with secondary antibodies for 1 h at room temperature and followed by a further triplicate wash. TBS and 0.05% (vol/vol) Tween 20 buffer were used to wash blots with phosphorylation events. Blots were scanned using a ChemiDoc MP Imaging System (Bio-Rad # #12003154).

### Immunoprecipitation (IP)

HEK293T cells were seeded at 3×10^6^ cells in 10-cm culture dishes and transfected 24 h later with a total of 8 µg plasmid using GeneJuice. The next day, supernatants were aspirated, and the cells were scraped on ice with 500 µl/well of cold IP lysis buffer as described previously [43,44]. Lysates were centrifuged at 17 000 ***g***/4 °C for 10 min using a cooling centrifuge (Thermo Scientific # 75002404). After centrifugation, 350 µl and 50 µl of cell lysates were transferred to pre-cooled IP and lysate control Eppendorf tubes, respectively. Lysate controls were frozen for later immunoblotting. Ten microlitres per sample of anti-FLAG M2 affinity gel beads (Sigma-Aldrich # A2220) were added into pre-cooled Eppendorf tubes, centrifuged at 3000 ***g*** for 1 min and then washed with cold IP lysis buffer. This step was repeated three times for the equilibration of anti-FLAG beads. Next, the beads (60 µl/sample) were mixed with lysis buffer and left for an overnight incubation at 4 °C. The samples were centrifuged at 3 000 ***g***/4 °C for 1 min and then washed with cold IP lysis buffer. The triplicate washing step was repeated and followed by the addition of 3 µg of FLAG tag peptide (Sigma-Aldrich # F3290) diluted with 80 μl of PBS (1 ×) per sample. The samples were rolled on a shaker at 4 °C for 30 min and then centrifuged at 3 000 ***g***/4 °C for 1 min for separation from the beads. Around 80 μl of each sample suspension was collected in pre-cooled Eppendorf tubes. Sample lysis buffer was added into controls (30 µl), IP samples (30 µl) and beads (50 µl) for immunoblotting with the indicated antibodies.

### Generation of MC089-expressing stable cell lines

HEK293T cells were seeded at 5×10^5^ cells ml^−1^ in six-well plates and transfected 24 h later with 4 µg of pMEP4-MC89-FLAG using GeneJuice. The next day, hygromycin B gold (InvivoGen # ant-hg) (200 μg ml^−1^) was added for the selection of transfected cells. The growth of selected cells was maintained with 100 µg ml^−1^ hygromycin B. Transfected cells were incubated with 1 µM of CdCl_2_ for 24 h to induce MC089 expression.

### ELISA

Supernatants from CdCl_2_-induced MEP4-MC089 HEK293T cells were assayed for IP-10/CXCL10 with an ELISA kit (R and D Systems # DY266-05) according to the manufacturer’s protocol. Optical density was measured at 450 nm using a Thermo Scientific Multiskan FC microplate photometer.

### IFN bioassay

HEK-blue IFN-α/β cells (InvivoGen # hkb-ifnab) were maintained with 30 µg ml^−1^ blasticidin (InvivoGen # ant-bl), 100 µg ml^−1^ zeocin (InvivoGen # ant-zn) and 100 µg ml^−1^ normocin (InvivoGen # ant-nr). A twofold serial dilution of recombinant human IFNβ standard (R and D Systems # 8499-IF-010/CF) was prepared using DMEM with 10% FBS, 1% penicillin and normocin (100 µg ml^−1^). Supernatants were harvested from CdCl_2_-induced MEP4-MC089 HEK293T cells and assayed for IFN secretion. Using flat-bottom 96-well culture plates, 80 µl of each standard and sample was added in triplicate with 120 µl/well of HEK-blue cells at 5×10^4^/well. After 24 h, 20 µl of induced HEK-blue supernatants was mixed with 180 µl QUANTI-blue (InvivoGen # rep-qb) in new flat-bottom 96-well culture plates. The plates were sealed with tin foil and incubated at 37 °C/5% CO_2_ for 15 min or until a blue-coloured reaction was observed. Secreted embryonic alkaline phosphatase (SEAP) levels were read at 620 nm using a Thermo Scientific Multiskan FC microplate photometer.

### Confocal microscopy

HEK293T cells were seeded at 8×10^4^ cells/well on glass chamber slides (Thermo Scientific # 154534) and transfected 24 h later using GeneJuice. MitoTracker deep red FM (Cell Signaling Technology # 8778) was prepared with DMSO according to the manufacturer’s specifications and added to the growth media to a final concentration of 200 nM for 30 min. Cells were then washed once with PBS (1 ×) and fixed with paraformaldehyde (PFA) diluted in PBS (Thermo Scientific # J19943K2) for 15 min. Following a triplicate wash with PBS, cells were blocked and permeabilized with 2% (wt/vol) BSA, 0.05% (vol/vol) Tween 20 and 0.3% (vol/vol) X-100 Triton diluted in PBS for 1 h. Cells were next incubated overnight in blocking buffer with the indicated antibodies: Alexa Fluor 488 FLAG tag antibody (1 : 1500), Alexa Fluor 647 HA tag antibody (1 : 500), anti-MAVS antibody (1 : 2000) and anti-TBKBP1 antibody (1 : 2000). The slides were washed with PBS three times and mounted with ProLong Gold Antifade reagent with DAPI (Invitrogen, P36931). Images were obtained using a Lecia SP8 confocal microscope with LAS X Life Science software.

### Affinity purification and mass spectrometry (AP-MS)

Four individual replicates of uninduced and CdCl_2_-induced MEP4-MC089 HEK293T cells (or pMEP4 empty vector controls) were cultured in 10-cm dishes for 24 h, then harvested and pelleted at 3 000 ***g***/4 °C for 5 min and snap-frozen using liquid nitrogen. Cell pellets were thawed on ice and resuspended in 1 ml ice–cold TAP lysis buffer [50 mM Tris-HCl, pH 7.5, 4.3% (vol/vol) glycerol, 0.2% (vol/vol) NP-40, 1.5 mM MgCl_2_, 100 mM NaCl] supplemented with EDTA-free complete protease inhibitor cocktail (Roche) and 250 U benzonase (Sigma-Aldrich). After incubation on ice for 30 min, lysates were centrifuged at 12 000 ***g*** for 5 min at 4 °C. Cleared lysates were incubated with 40 µl anti-Flag M2 affinity gel (Sigma-Aldrich) for 1 h on a rotating wheel at 4 °C. After incubation, the resin was washed in TAP lysis buffer (with the final two washes performed without NP-40) and resuspended in 40 µl 6 M guanidinium-HCl in 100 mM Tris, pH 8.5, supplemented with 10 mM tris(2-carboxyethyl)phosphine and 40 mM chloroacetamide. After 30 min of incubation at room temperature in the dark, lysates were diluted 1 : 10 with digestion buffer (10% acetonitrile, 25 mM Tris, pH 8.5) and incubated with 0.5 µg EndoLysC (Wako Chemicals) and 0.5 µg sequencing-grade modified trypsin (Promega) overnight on a rotating wheel at room temperature. After digestion, peptides were acidified with trifluoroacetic acid, desalted on reversed-phase C_18_ StageTips, and eluted before liquid chromatography-tandem mass spectrometry (LC-MS/MS) using buffer B (80% acetonitrile and 0.5% acetic acid). Eluted peptides were analysed on a nanoflow EASY-nLC system coupled to an LTQ-Orbitrap XL mass spectrometer (Thermo Fisher Scientific). Peptide separation was achieved on a C_18_ reversed-phase column (ReproSil-Pur C_18_-AQ; 1.9 µm by 200 mm by 0.075 mm; DrMaisch), using a 120-min linear gradient from 2 to 60% acetonitrile in 0.1% formic acid. The LTQ-Orbitrap XL MS was operated with a Top10 MS/MS spectrum acquisition method in the linear ion trap mode for each MS full scan. Raw files were processed with MaxQuant (version 1.4.1.4) and searched with the built-in Andromeda search engine against a human protein database (UniprotKB, release 2012_01) concatenated with a decoy of reversed sequences, using a label-free quantification (LFQ) algorithm as described previously [7]. Carbamidomethylation was set as a fixed modification, while methionine oxidation and protein N-acetylation were included as variable modifications. The search was performed with an initial mass tolerance of 6 ppm for the precursor ion and 0.5 Da for the fragment ions. Search results were filtered in Perseus (version 1.4.1.8), with a false discovery rate (FDR) of 0.01. Prior to statistical analysis, known contaminants and reverse hits were removed. Proteins identified with at least two unique peptides and a minimum of 2 quantitation events in at least one experimental group were considered for analysis. LFQ protein intensity values were log-transformed, and missing values were filled in by imputation, with random numbers drawn from a normal distribution. Significant interactors were determined using the two-sample *T*-test with the Welch correction after 250 permutations, with the FDR threshold set to 0.01 and *S*_0_ empirically set to 1. Results were plotted using R (volcanoseR release version V1.0.3).

### Statistical analysis

GraphPad PRISM 8 was used for statistical analysis and generation of graphs. Statistical significance was measured using unpaired student’s *T*-tests and presented as *P<0.05, **P<0.01, ***P<0.001 and ****P<0.001.

### Data availability statement

The mass spectrometry proteomic data have been deposited to the ProteomeXchange Consortium via the PRIDE partner repository with the dataset identifier PXD054178. The data can be accessed with the following reviewer login information: Username: reviewer_pxd054178@ebi.ac.uk; Password: tckpUqsaICx0. Alternatively, a reviewer can also log in to the PRIDE website using the following details: Project accession: PXD054178, Token: AFa0vwdSszDT.

## Results

### MC089 is a specific inhibitor of IRF-dependent gene induction by nucleic acid-sensing pathways

**Fig. 1.**
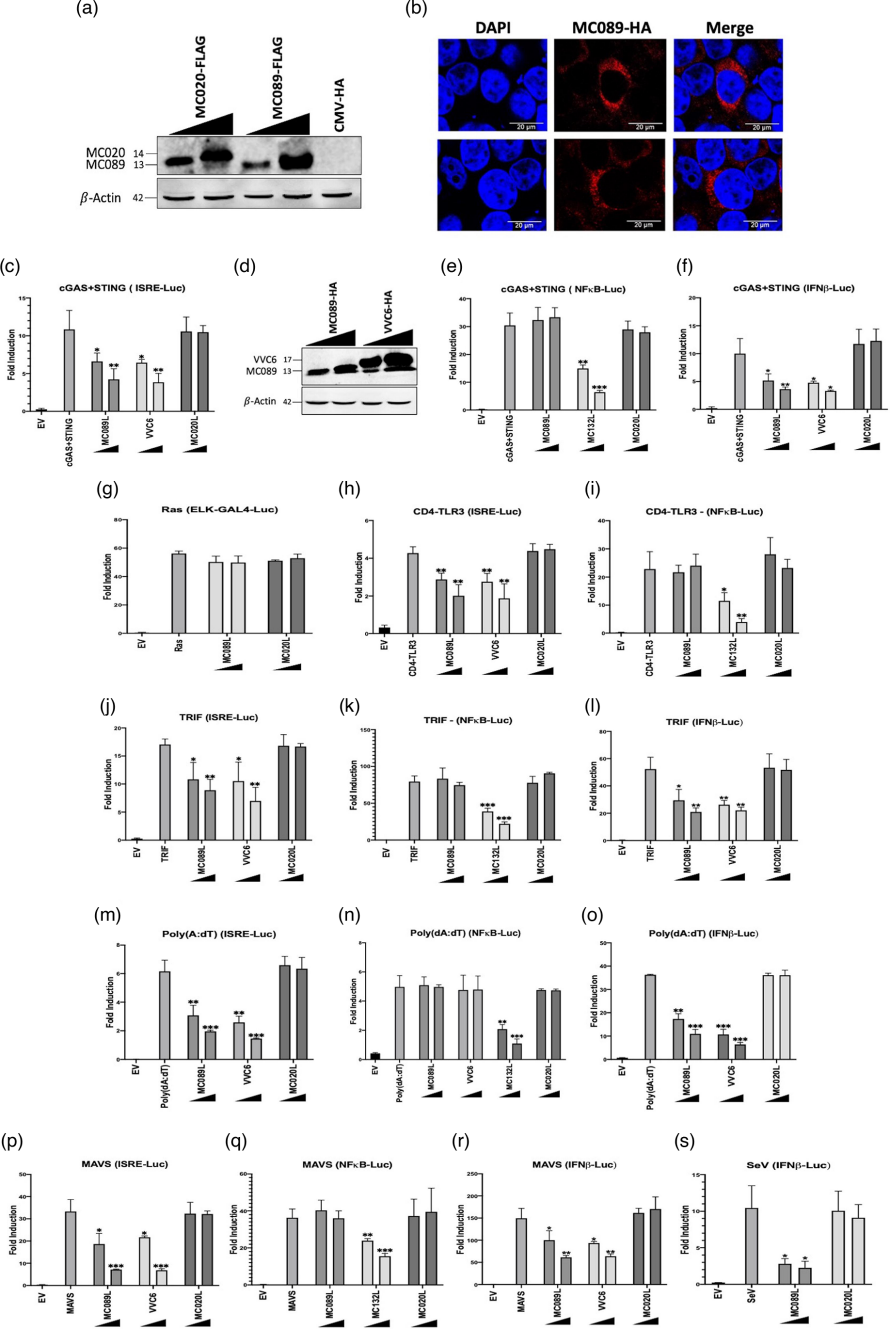
MC089 is a specific inhibitor of IRF-dependent gene induction by nucleic acid-sensing pathways. (a and c) HEK293T cells were seeded at 5×10^5^ cells/well in six-well plates and transfected 24 h later with 3 μg empty vector (pCMV-HA), 1.5 μg or 3 μg (equivalent to amounts used in the luciferase assay) pCEP4 constructs expressing MC089-FLAG and MC020-FLAG (**a**) or MC089-HA and VVC6-HA (**d**). Cell lysates were harvested and western blotted with the indicated antibodies. (**b**) Confocal localization of MC089 in HEK293T cells. Cells were transfected with 500 ng of pCEP4-MC089-HA expression vector, fixed 24 h later and stained with HA tag antibody (red) and DAPI (blue). (c, e–s) HEK293T cells were seeded at 2×10^5^ cells ml^−1^ and transfected 24 h later with 80 ng of NF-κB, ISRE, IFNβ or pFR-luciferase (GAL4-responsive) reporter. For GAL4-responsive assay, 5 ng of pFA-Elk1 was co-transfected with pFR-luciferase. To normalize firefly luciferase, 40 ng of pGL3-*Renilla* control was utilized. Cells were transfected with 25 ng or 50 ng pCEP4 vectors of the indicated MCV ORFs or VVC6. Activators of the pathways were employed accordingly: cGAS (25 ng) and STING (25 ng), RasHa (50 ng), CD4-TLR3 (50 ng), TRIF (10 ng), Poly(dA:dT) (1 μg ml^−1^), MAVS (10 ng) or SeV. The empty vector (indicated by EV) was used as a control of pathway activation. The total amount of DNA was adjusted to a final volume of 220 ng using the empty vector control (pCMV-HA). Cell lysates were harvested and assayed for luciferase activity. Schematics are representative of three or more individual experiments. Firefly luciferase activity was normalized to *Renilla* luciferase activity, and data are presented by fold induction. Bars indicate mean±standard deviation. Statistical significance is denoted as *P<0.05,**P<0.01 and ***P<0.001.

We previously screened a library of MCV ORFs for their ability to inhibit innate antiviral signalling pathways using a high-throughput luciferase assay-based screening system and identified MC089 as an inhibitor of cGAS–STING-induced activation of an IRF-responsive ISRE reporter but not an NF-κB-responsive reporter (data not shown). MC089 is a 114-amino acid protein (predicted size 13 kDa) encoded by the MC089L ORF located on the left-hand terminus of the MCV genome [46]. It shows homology with the Fowlpox virus protein FPV145 sharing approximately 28% sequence identity [47] and a 56% similarity to the hypothetical protein EMCLV086L from the recently sequenced equine molluscum contagiosum-like virus genome [48]. We previously used MC020, a 139-amino acid MCV protein with a predicted size of 14 kDa, and no inhibitory activity on any innate or control signalling pathways tested, as a negative control [49]. Thus, MC020 was also used as a negative control for MCV protein in these experiments. Equivalent expression of MC089 and MC020 was detected by immunoblotting (Fig. 1a). Confocal analysis showed that MC089 displays distinctive punctate distribution in the cytoplasm of expressing cells (Fig. 1b).

To analyse the effect of MC089 on innate immune signalling pathways in more detail, we employed a luciferase assay-based screening system, driving activation of virus-sensing pathways at defined points with PRR agonists, viral infection or overexpressed innate signalling proteins and measuring the effect of MC089 on activation of three distinct luciferase reporter constructs: κB-luciferase, ISRE-luciferase and IFNβ promoter-luciferase. While the κB luciferase reporter measures NF-κB activity and the ISRE luciferase reporter measures IRF (primarily IRF3) activity in primary sensing pathways [9], both NF-κB and IRFs regulate the activation of the natural IFNβ promoter reporter [6]. However, IRFs have a more dominant role in regulating the IFNβ promoter due to potent ISRE sites in two promoter regions (PRDI and PRDIII) [50,54].

The cGAS–STING DNA-sensing pathway can be reconstituted in cGAS and STING co-transfected HEK293T cells where both proteins are not normally expressed in these cells [55,56]. As an inhibitory reference for MC089 inhibition of these pathways, we used the Vaccinia virus IRF activation inhibitor C6 (VVC6), which binds TBKBP1, NAP1 and TANK to inhibit TBK1 and IKKε activation [45]. The MCV inhibitor of NF-κB activation MC132 was used as a positive control for the inhibition of NF-κB luciferase activity [43]. Analogous to VVC6, while MC089 expression potently inhibited IRF and IFNβ promoter activation by this system, it had no effect on cGAS–STING-induced NF-κB activation (Fig. 1c, e, f). Additionally, MC089 did have inhibit basal reporter activity (Fig. S1A, available in the online Supplementary Material). Interestingly, MC089 was substantially more potent in inhibiting IRF and IFNβ activation with lower levels of expression than VVC6 (Fig. 1d). MC089 specificity for IRF inhibition was further confirmed by the absence of an inhibitory effect on the Elk1 mitogenic signalling pathway driven by RasVHa, which was parsed using an Elk-1-GAL4 fusion promoter luciferase construct (Fig. 1g).

We next examined the effect of MC089 on the dsRNA-sensing TLR3 pathway by overexpressing a constitutively active chimeric construct of the CD4 signalling domain and TLR3 or by overexpressing TLR3’s primary adapter TRIF [57]. Consistent with our other observations, MC089 did not affect NF-κB activation by CD4-TLR3 but inhibited ISRE activation by the same activator (Fig. 1h, i). Similarly, while MC089 had no effect on TRIF-driven NF-κB activation, both MC089 and VVC6 inhibited ISRE activation and that of the IFNβ promoter (Fig. 1j–l).

The RIG-I pathway has been shown to indirectly recognize double-stranded DNA (dsDNA) in HEK293T cells through the transcription of AT-rich dsDNA into the 5′-triphosphate RNA ligand of RIG-I by the enzyme RNA Pol III [58]. Thus, the stimulation by synthetic dsDNA ligand, Poly(dA:dT), and overexpression of the downstream regulator of RIG-I signalling, MAVS, were used to activate the RIG-I pathway. Again, MC089 and VVC6 inhibited activation of ISRE by this pathway and IFNβ promoter activity without affecting NF-κB activity (Fig. 1m–r). We next examined the effect of virus-induced IFN-β promoter activity. Indeed, the RIG-I-activating virus SeV induces IFNβ promoter activation in HEK293T cells, which was impaired through MC089 expression (Fig. 1s). Together, these data suggested that MC089, like VVC6, is a specific inhibitor of IRF activation and targets a downstream point common to multiple innate virus-sensing pathways.

### MC089 inhibits IRF3-dependent gene expression at the level of IRF-activating complexes

**Fig. 2.**
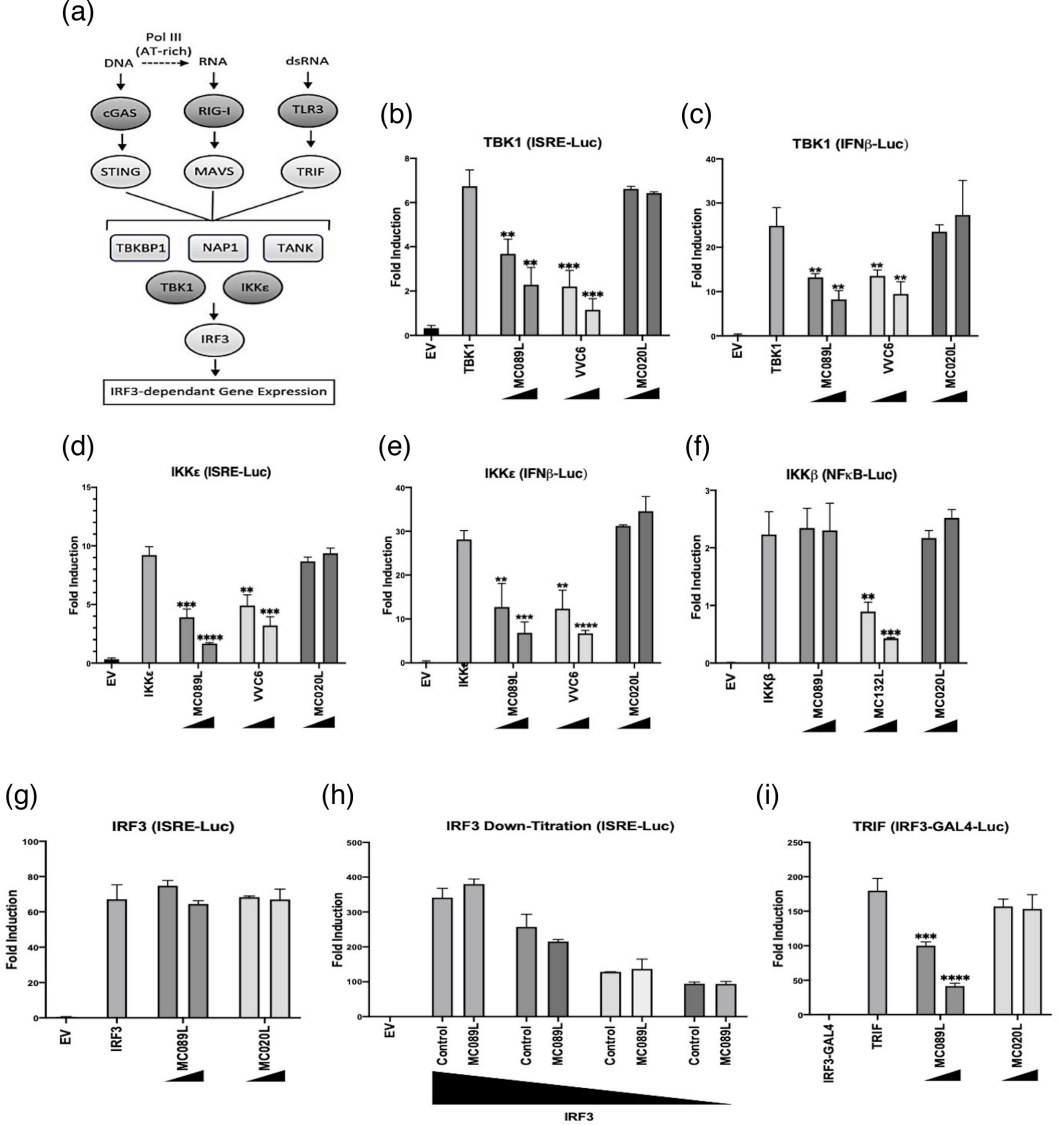
MC089 inhibits IRF-dependent gene expression at the level of IRF3-activation complexes. (**a**) Summary of IRF3-activating pathways. (**b–g**) HEK293T cells were seeded at 2×10^5^ cells ml^−1^ and transfected 24 h later with 80 ng of ISRE, IFNβ or NF-κB luciferase reporter. To normalize firefly luciferase, 40 ng of pGL3-Renilla control was used. Cells were transfected with 25 ng or 50 ng pCEP4 vectors of the indicated MCV ORFs or VVC6. Activators of the pathways were added accordingly: TBK1 (50 ng), IKKε (50 ng), IKKβ (25 ng) or IRF3 (50 ng). The total amount of DNA was adjusted to a final volume of 220 ng with the empty vector control (pCMV-HA). The empty vector (indicated by EV) was used as a control of pathway activation. Cell lysates were harvested and assayed for luciferase activity. Data were normalized to the empty vector and presented by percentage activity compared with the positive control. Bars indicate mean±standard deviation. Statistical significance is denoted as **P<0.01, ***P<0.001 and ****P<0.0001. (**h**) Similarly, HEK293T cells were seeded at 2×10^5^ cells ml^−1^ and transfected 24 h later with 80 ng of ISRE luciferase reporter and 40 ng of pGL3-Renilla control. IRF3 doses used were 25, 10, 5 and 3 ng, respectively. The total amount of DNA was adjusted to a final volume of 220 ng using the empty vector control (pCMV-HA). (**i**) Similar to b–g, but cells were transfected with pFR-luciferase reporter (80 ng) and IRF3-GAL4 (5 ng) and stimulated as indicated with TRIF (10 ng). Cell lysates were harvested and assayed for luciferase activity. Firefly luciferase activity was normalized to *Renilla* luciferase activity, and data are presented by fold induction. Bars indicate mean±standard deviation. Assay data are representative of triplicate individual experiments.

All upstream virus-sensing pathways lead to the activation of IRF-activating complexes consisting of scaffold proteins (TBKBP1, NAP1 or TANK), which regulate the activity of the kinases TBK1 and IKKε, which in turn phosphorylate and thus activate IRF3 to drive gene expression (Fig. 2a). To further clarify the point of inhibition of MC089 on IRF-activating pathways, we next examined the effect of MC089 on signalling by the direct activators of IRFs, TBK1 and IKKε. As VVC6 is known to inhibit IRF3 activation by binding to the IRF kinase scaffold proteins TANK, NAP1 and TBKBP1, it was used again as an inhibitory reference [45]. Comparable to VVC6, MC089 inhibited both TBK1- and IKKε-dependent ISRE and IFNβ promoter activation (Fig. 2b–e). Consistent with previous data, MC089 did not affect IKKβ activation of NF-κB (Fig. 2f). Interestingly, MC089 was substantially more potent than VVC6 at inhibiting IKKε-induced ISRE activation (Fig. 2d). We also attempted to drive ISRE luciferase activity by the scaffold proteins of the IRF3-activation complex but did not observe an activation of the system using these proteins (Fig. S1b–d). This was not unexpected, as we have previously observed that overexpression of some signalling components does not function to drive the activation of the system.

We next investigated the effect of MC089 on direct activation of ISRE by overexpression of IRF3. In our previous work on the MCV inhibitor MC132, which directly targets the NF-κB p65 subunit for degradation, we found that, while MC132 could inhibit activation of κB-luciferase, this effect was bypassed at higher levels of p65 overexpression [43]. Thus, the titration of transcription factor activators of reporter systems is a necessary step in confirming inhibitors target upstream and not at the level of the transcription factor itself. Consistent with inhibition at a point upstream of IRF3, overexpression of this transcription factor bypassed ISRE reporter inhibition by MC089 (Fig. 2g). MC089 did not inhibit IRF3-activated ISRE, even when transfecting low amounts of IRF3-expressing vector (Fig. 2h), excluding saturation effects and suggesting that MC089 was targeting upstream activating complexes rather than the IRF3 transcription factor directly. Additionally consistent with a hypothesis of inhibiting activation of IRF3 activation at the level of upstream kinases, MC089, but not MC020, inhibited TRIF-mediated IRF3-GAL4 transactivation (Fig. 2i).

### MC089 selectively associates with MAVS, IKKε, TBKBP1 and NAP1

**Fig. 3.**
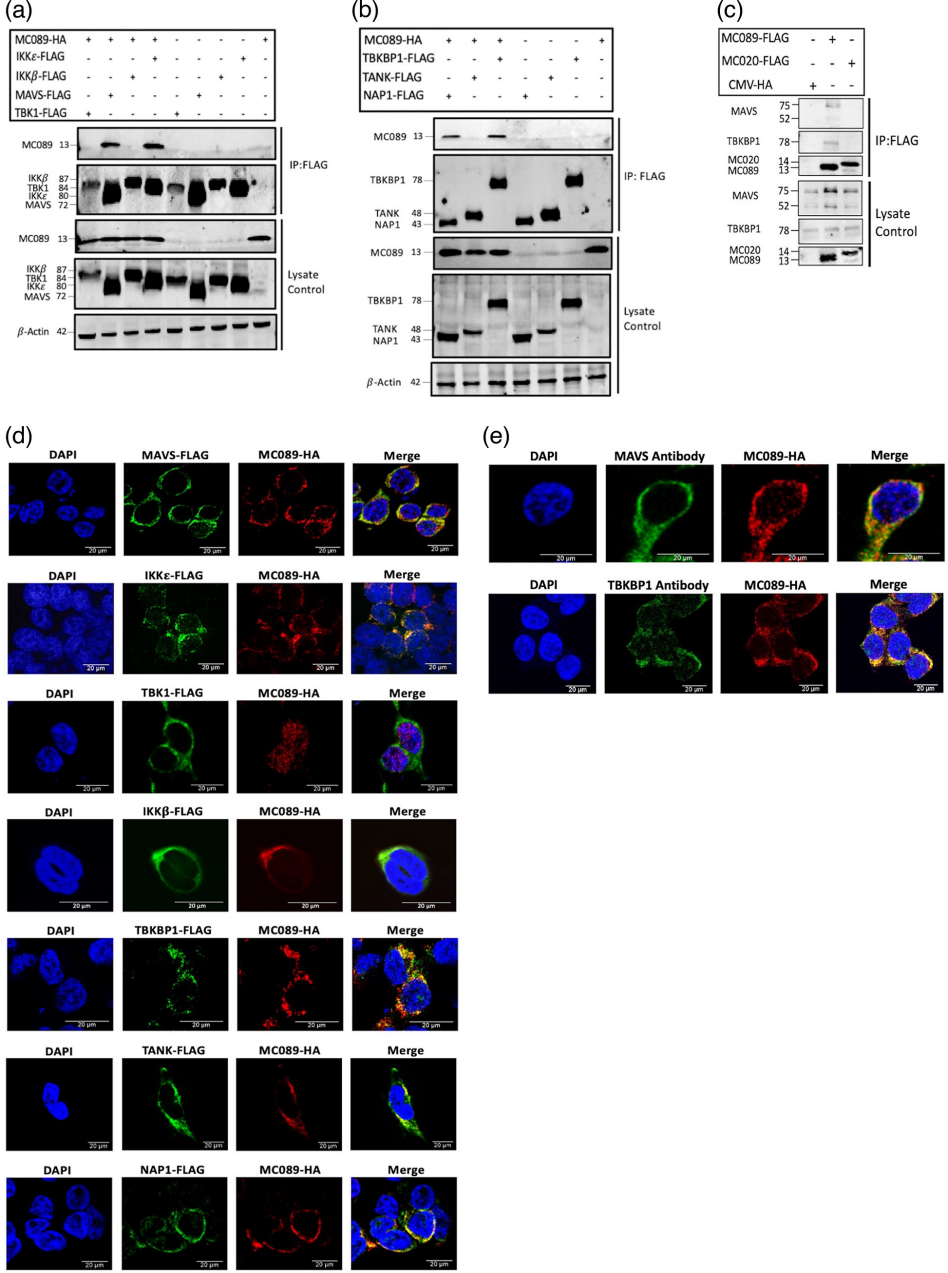
MC089 selectively associates with MAVS, IKKε, TBKBP1 and NAP1. (a and b) HEK293T cells were seeded at 3×10^6^ cells/culture dish and transiently transfected 24 h later with a total of 8 μg of pCEP4-MC089-HA and the indicated signalling pathway component FLAG-tagged plasmids. After 24 h, cell lysates were immunoprecipitated using anti-FLAG M2 affinity gel beads, eluted with FLAG tag peptide and probed with the appropriate antibodies: (a and b) anti-HA (first and third panels), anti-FLAG (second and fourth panels) and anti-β-actin (fifth panel). (**c**) Similarly, pCEP4-MC089-HA was immunoprecipitated with endogenous MAVS antibody or TBKBP1 antibody. (**d**) HEK293T cells were seeded at 8×10^4^ cells/well on glass chamber slides and transiently transfected 24 h later with a total of 500 ng of pCEP4-MC089-HA and the indicated signalling pathway component FLAG-tagged plasmids. Cells were fixed 24 h later and stained with HA tag antibody (red), FLAG tag antibody (green) and DAPI (blue). (**e**) pCEP4-MC089-HA (red) colocalization with MAVS antibody (green) or TBKBP1 antibody (green). Data are representative of triplicate experiments.

We next investigated the possible association of MC089 with known constituent proteins of the IRF3 activating complexes. The NF-κB-regulating kinase IKKβ was used as a negative control for MC089 in co-immunoprecipitation experiments due to its lack of involvement in IRF activation while having a high similarity to IRF-activating members of the IKK family. Although no association was detected with TBK1 or IKKβ, MC089 interacted with IKKε and MAVS (Fig. 3a). We next probed the association of MC089 with the IRF-activating complex adapter proteins TBKBP1, TANK and NAP1. These adapters provide a platform for the assembly of IRF-activating kinases for their subsequent phosphorylation of IRF3 [34,37]. Although no association was detected with TANK, MC089 interacted with TBKBP1 and NAP1 (Fig. 3b). Conversely, MC020 showed no interaction with IKKε, MAVS, TBKBP1 or NAP1 (Fig. S2). The interaction of MC089 with selected endogenous proteins, MAVS and TBKBP1, was also confirmed in HEK293T cells using cognate antibodies for these proteins (Fig. 3c).

We also probed the cellular localization of IRF-activating complex proteins and MC089 by confocal microscopy to investigate if MC089 and its immunoprecipitated proteins are co-localized in close proximity within the cell for a possible interaction to occur. Consistent with co-immunoprecipitation data, strong co-localization was observed for MC089 with IKKε, NAP1 and TBKBP1 with partial co-localization with MAVS (Fig. 3d). Also consistent with co-immunoprecipitation data, such strong co-localization between MC089 and TBK1 IKKβ or TANK was not observed. Curiously, when MC089 and TBK1 were co-expressed, MC089, normally cytoplasmic, consistently displayed nuclear staining. Additionally, as for co-immunoprecipitation experiments, we confirmed the co-localization of MC089 with endogenous MAVS and TBKBP1 in HEK293T cells (Fig. 3e).

### MC089 associates with mitochondria

**Fig. 4.**
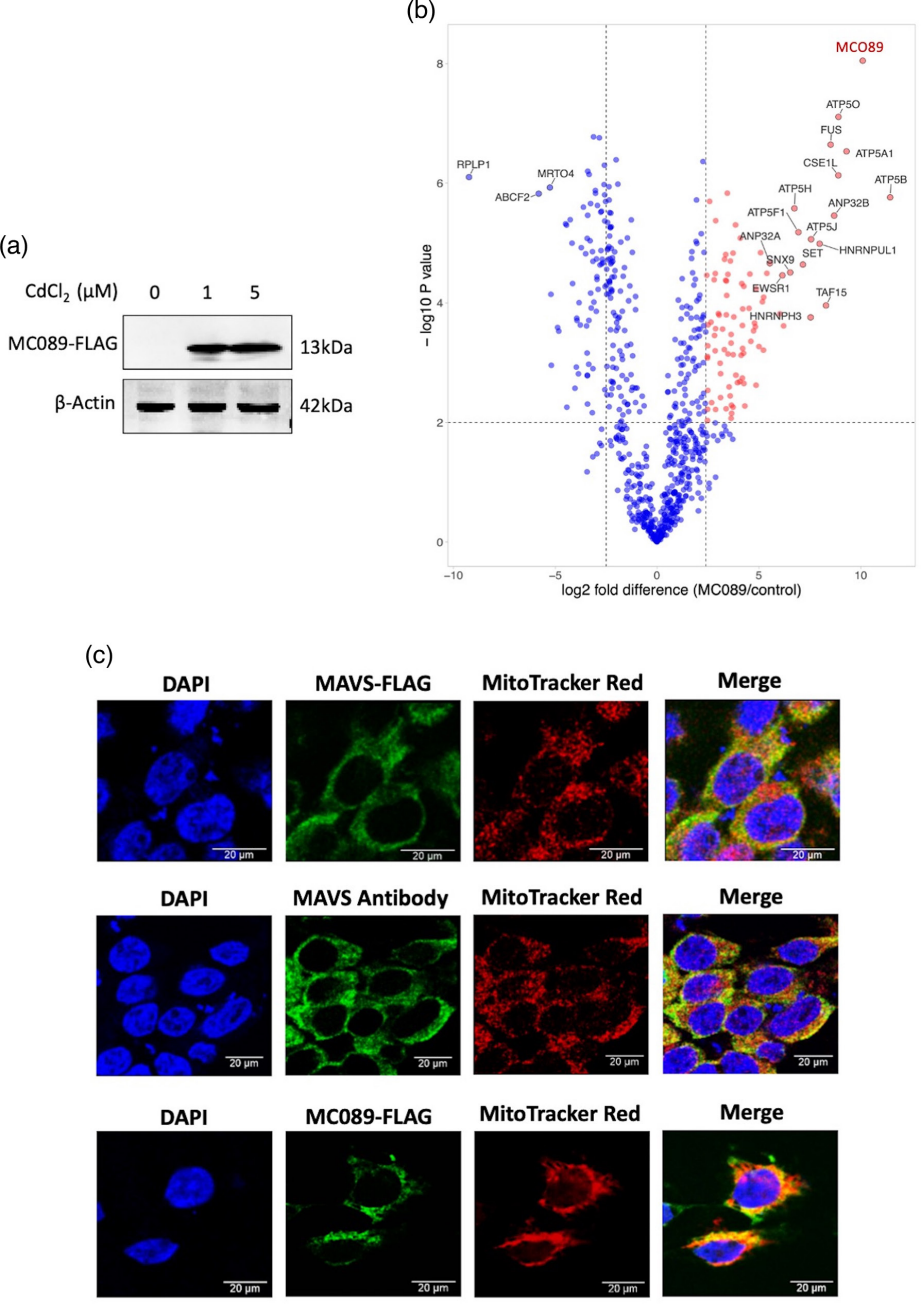
MC089 associates with mitochondria. (**a**) HEK293T cells were stably transfected with MEP4-MC089-FLAG vector and treated with CdCl_2_ at the indicated concentrations to induce MCV protein expression. Cell lysates were harvested for western blot and probed with an anti-Flag antibody. (**b**) Volcano plot of MC089-interacting proteins from MC089-expressing HEK293T cells (versus empty vector-expressing cells) using AP-MS. The volcano plot displays proteins above the significance threshold co-purifying with immunoprecipitated MC089 (highlighted in red) (*n*=4). (**c**) Mitochondrial confocal localization of MAVS and MC089. HEK293T cells were seeded at 8×10^4^ cells/well on glass chamber slides and transfected 24 h later with a total of 500 ng of MAVS-FLAG (upper panel), pCMV-HA empty vector (middle panel) or pCEP4-MC089-FLAG (lower panel). After 24 h, MitoTracker deep red FM diluted in DMSO was added to the growth media at 200 nM for 30 min. Cells were fixed and stained with DAPI (blue), FLAG tag antibody (green) or MAVS antibody (green). Data are representative of triplicate experiments.

To further probe the inhibitory mechanism of MC089, we sought to identify co-purifying proteins through unbiased AP-MS. We generated a stable inducible HEK293T cell line expressing MC089-FLAG using the CdCl_2_-inducible pMEP4 vector containing the MC089 ORF with a C-terminal FLAG tag. We confirmed CdCl_2_-inducible expression of MC089-FLAG in this cell line (Fig. 4a). AP-MS analysis of MC089 co-purifying proteins revealed a significant enrichment of mitochondrial proteins including multiple mitochondrial ATP synthase subunits (ATP5J, ATP5O, ATP5H, ATP5B, ATP5F1 and ATP5A1) [59,60] (Fig. 4b). This suggested that MC089 associates with mitochondria, which was intriguing given the prior finding of immunoprecipitation and co-localization of MC089 with MAVS, a protein known to associate primarily with mitochondria and partially with endoplasmic reticulum (ER) and peroxisomes [61,64]. IKKε, NAP1, TBKBP1 and MAVS were likely not observed as proteins co-purified with MC089 in AP-MS due to their relatively low abundance in HEK293T cells with the overrepresentation of more highly expressed mitochondrial proteins that were heavily enriched in pull-down material.

To confirm the mitochondrial association of MC089, we stained mitochondria using MitoTracker red in MC089 and MAVS-expressing cells and examined cells by confocal microscopy. We first confirmed that both overexpressed MAVS-FLAG and endogenous MAVS partly associate with mitochondria in HEK293T cells and that MC089 displays a similar pattern of partial association with mitochondria (Fig. 4c).

### MC089 inhibits IRF3 phosphorylation (Ser396) and IRF3-dependent cytokine production

**Fig. 5.**
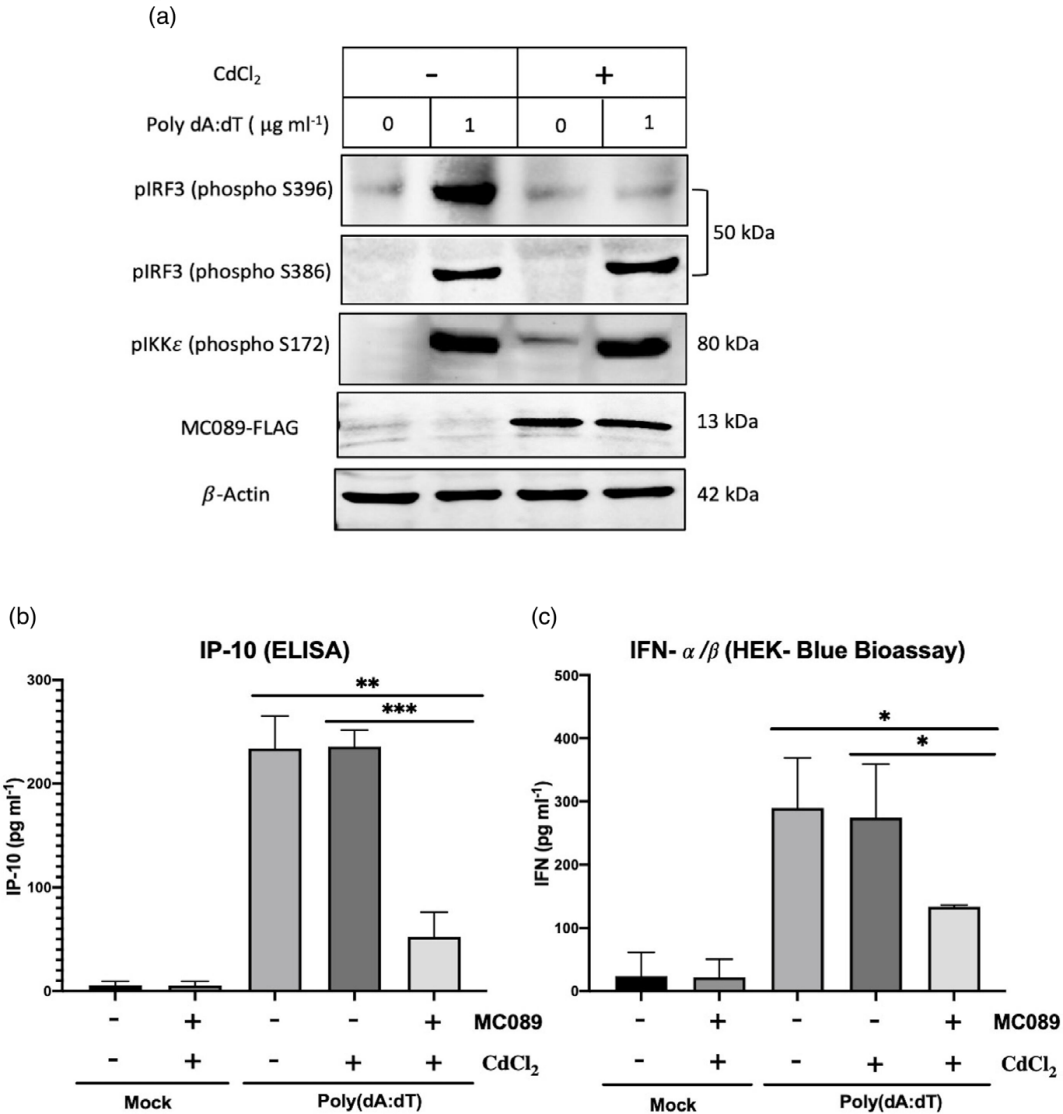
MC089 suppresses IRF3 phosphorylation (Ser396) and TI-IFN secretion. (**a**) HEK293T cells were stably transfected with MEP4-MC089-FLAG vector and treated with 1 μM of CdCl_2_ to induce MCV protein expression. After 24 h, cells were stimulated with 1 μg ml^−1^ of Poly(dA:dT) for 16 h to induce protein phosphorylation. Cell lysates were harvested for western blot and probed with the indicated antibodies. Blots are representative of triplicate experiments. (b and c) MEP4-MC089-expressing HEK293T supernatants were harvested and assayed for IP-10 ELISA (**b**) or IFN-α/β detection (**c**). Cells were stimulated 1 μg ml^−1^ of Poly(dA:dT) for 16 h, with mock-transfected cells (mock) serving as a negative control for cytokine secretion. Concentrations were calculated from standard curves generated from measured optical density. Data are representative of triplicate experiments. Bars indicate mean±standard deviation. Statistical significance is denoted as *P<0.05, **P<0.01 and ***P<0.001.

Having identified MC089 as a novel MCV inhibitor of ISRE promoter activation, interacting with IKKε, NAP1, TBKBP1 and MAVS, partly at the level of mitochondria, we next sought to further understand how association with these proteins might inhibit IRF3 activation and the downstream consequences on IRF3-dependant gene expression. To do this, we examined Poly(dA:dT)-stimulated phosphorylation of two key residues on IRF3, serine 386 and serine 396, known to be critical for IRF3 activation and transactivation [40]. Interestingly, while MC089 inhibited Poly(dA:dT)-induced phosphorylation of IRF3 serine 396, it had no effect on serine 386 phosphorylation. The interaction between MC089 and IKKε raised the possibility that it may block IKKε activation, which can be assessed by IKKε phosphorylation at serine 172. To investigate this, we probed the phosphorylation of IKKε at the serine residue 172 and found that MC089 had no effect. This suggested that MC089 prevents pIRF3 Ser396 independently of IKKε phosphorylation (Fig. 5a).

We then assessed the effect of MC089 on Poly(dA:dT)-activated IRF3-dependent gene expression, specifically IP-10 and TI-IFNs. Like IFNβ, IP-10 is known to be a strongly ISRE- and IRF-regulated gene [65,67]. To assess if MC089 inhibition of IRF3 activity inhibits the production of TI-IFNs, we measured IFN-α/β levels in the supernatants of MC089-expressing HEK293T cells after Poly(dA:dT) stimulation using HEK-blue IFN-α/β bioassay reporter cells, which possess a SEAP reporter under the control of the IFN-stimulated gene 54 (ISG54) promoter. We observed that MC089 expression inhibited both Poly(dA:dT)-induced IP-10 and TI-IFN production (Fig. 5b, c).

**Fig. 6.**
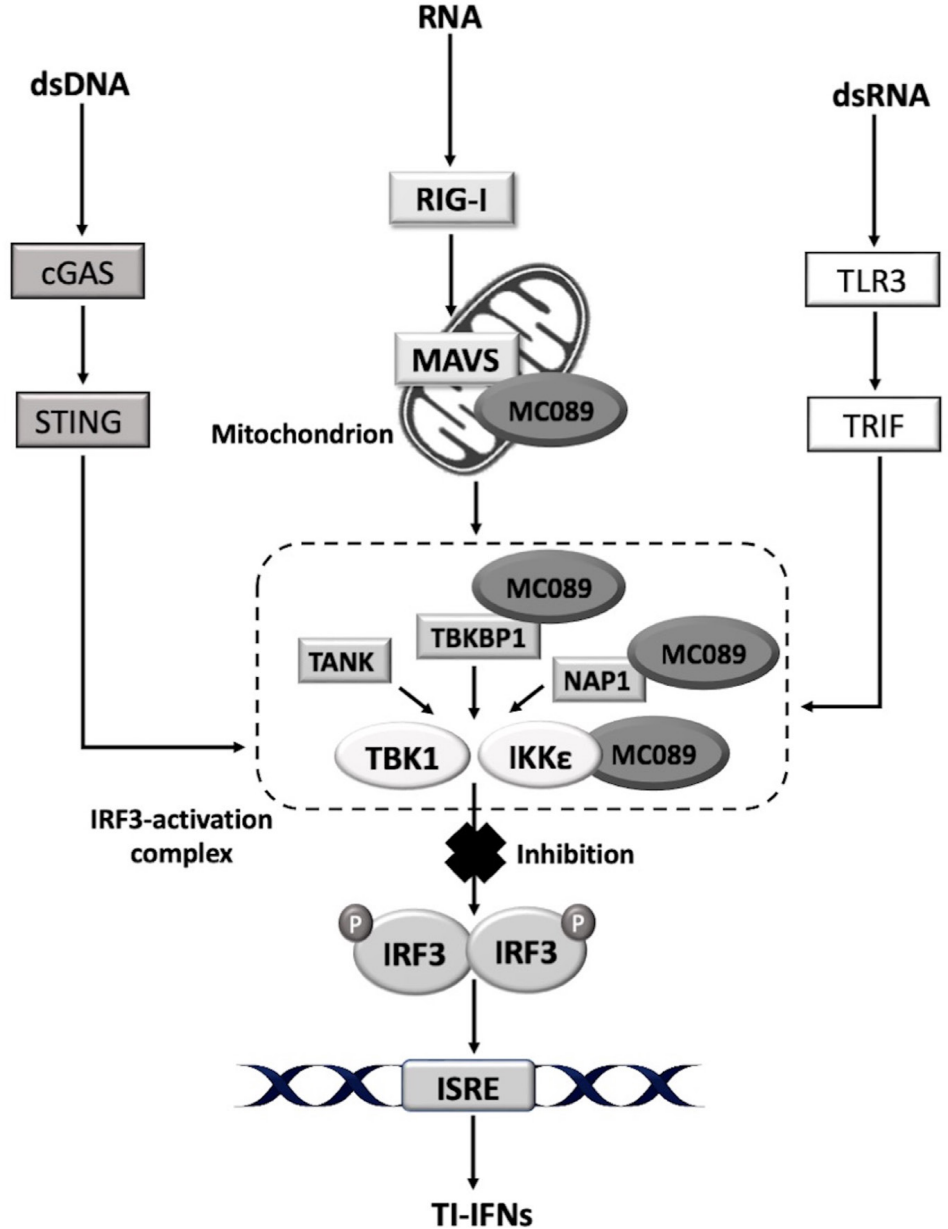
Model of MC089-mediated inhibition of IRF3 activation. Upon sensing viral nucleic acids, multiple signalling pathways drive the activation of IRF3 via phosphorylation and ubiquitination of a series of adaptor proteins and kinases. MC089 interacts with the kinase IKKε and its scaffold proteins TBKBP1 and NAP1 at the level of the IRF3-activation complex. It also impedes the RIG-I-sensing pathway by localizing to the mitochondria and associating with MAVS. Consequently, MC089 blocks IRF3 activation by inhibiting phosphorylation at serine 396; thus, it prevents the subsequent induction of TI-IFNs.

Taken as a whole, these data show that MC089 is a novel, specific MCV inhibitor of IRF3 activation, the first inhibitor specific to IRF3 activation to be discovered in MCV. We propose a model whereby MC089 inhibits through association with upstream activating complexes containing MAVS, IKKε, TBKBP1 and NAP1, partly in association with mitochondria to inhibit specific phosphorylation events needed for IRF3 activation (serine 396), thus inhibiting gene expression from IRF3-dependent genes like IP-10 and TI-IFNs (Fig. 6).

## Discussion

MCV is the only known extant human-specific poxvirus, which appears to have evolved for human infection. It causes benign skin lesions typically absent of inflammation with a long duration to clearance [3]. The human-specific nature of MCV, duration of infection and mild presentation of its lesions suggest that MCV possesses highly effective innate immune inhibitors like all other well-characterized poxviruses. Unfortunately, attempts to culture MCV *in vitro* and infect cells in culture have not been successful, which somewhat restricts the investigation of host–pathogen interactions [68,69]. Nevertheless, several novel MCV-derived inhibitors have been discovered through the characterization of MCV ORFs in isolation. We previously identified and characterized the inhibitory mechanisms of three novel inhibitors MC132, MC005 and MC008, which target the NF-κB pathways [43,44, 49].

Here, we report the discovery of MC089 as a novel MCV inhibitor of IRF3 activation by both DNA- and RNA-sensing pathways. MC089 appears to inhibit IRF3 activation through interaction with specific complexes containing IKKε, TBKBP1, NAP1 and MAVS, the latter at least partly in association with mitochondria. Two MCV inhibitors of IRF3 activation have been previously described in MCV, MC159 and MC160 [17]. While the mechanism of MC160 is still unclear, MC159 binds both TBK1 and IKKε, distinct from the specificity of MC089 binding only IKKε [70]. As both MC159 and MC160 also inhibit the NF-κB pathways [70,71], this makes MC089 the first MCV inhibitor to specifically target IRF-activating pathways and of particular interest for future study.

MC089 showed a similar pattern of inhibition to another specific poxvirus inhibitor of IRF activation, the VVC6 protein which interacts with the scaffold proteins TANK, TBKBP1 and NAP1 to suppress IRF3 activity [45]. This indicated that MC089 might follow a similar mode of targeting IRF3 activation at the level of the IRF3-activation complex. However, MC089’s ability to inhibit the system at lower levels of expression suggested a much more potent inhibitory mechanism where multiple key proteins of IRF3 pathways are involved. Additionally, MC089 was significantly more potent than VVC6 at inhibiting IKKε-induced ISRE activation, which supports the association between MC089 and IKKε compared with VVC6, which does not directly interact with either IKKε or TBK1.

The inhibition of both cGAS–STING DNA-sensing pathways and the TLR and RLR RNA-sensing pathways may be explained by MC089 targeting of IKKε as well as TBKBP1 and NAP1. However, MC089 also appears to specifically target the RLR pathways by association with MAVS, at least partly at the level of mitochondria as indicated by MC089 co-purifying with mitochondrial proteins and co-localization with both MAVS and mitochondria in HEK293T cells. It is also worth noting that there is considerable crosstalk between STING and RLR pathways, suggesting that MAVS interaction might also contribute to MC089 inhibition of cGAS–STING-mediated IRF3 activation. For example, the ssRNA genome of the Japanese encephalitis virus is recognized by RIG-I, which then recruits STING to initiate a downstream cascade leading to the antiviral response in neurons [72]. In several studies, STING appeared to interact with RIG-I and MAVS, in a complex that was stabilized upon virus infection [72,74]. Indeed, a role for STING in RNA-sensing pathways is further indicated by the fact that many RNA viruses have evolved STING inhibitory strategies, including hepatitis C virus, influenza A virus and dengue virus [75,78]. Additionally, in HeLa or HepG2 cells, TBK-1 phosphorylation after DNA transfection or DNA virus infection required MAVS–TBK1 interaction and MAVS knockdown in these cells markedly reduced phospho-TBK1 and IFN-β levels induced by cytoplasmic DNA [79].

Despite the lack of interaction of MC089 with TBK1, the inhibition of TBK1-driven IRF activation could be explained by its targeting of TBKBP1 and NAP1. These scaffold proteins along with TANK are essential for the dimerization and auto-phosphorylation of IRF3 kinases, recruiting them at a specific cellular site [37,80]. Although the exact mechanism by which IRF3 kinases interact with their scaffold proteins is still not fully understood [31], all three scaffold proteins target the same binding site on the kinases, suggesting a competitive mode of binding and signal transduction specificity (102). Viruses evolving specific inhibitors of the three scaffold proteins reflect their importance in IRF3 regulation. The non-human-adapted vaccinia virus encodes the protein C6, which inhibits IRF3 activation, without affecting NF-κB activation, by interaction with TBKBP1, NAP1 and TANK [45]. MC089 interaction with TBKBP1 and NAP1, but not TANK, could give new insights into the roles and subcellular localization of these scaffold proteins. TANK has been discovered to migrate from its perinuclear localization to autophagy-related vesicles upon DNA stimulation [37], while NAP1 and TBKBP1 have been reported to have punctate subcellular localization, and they have been also partially detected at the Golgi compartments [37,81]. As MC089 also displays punctate distribution and partial association with mitochondria, this along with its interaction with MAVS may indicate a mitochondrial functional specificity for TBKBP1 and NAP1. It might also suggest that MC089 uses TBKBP1-NAP1 to recruit IKKε to MAVS in the mitochondria.

Additionally, MC089 targeting of both MAVS and IKKε may give insights into their association with virus infection. MAVS has been found to bind IKKε through its C-terminal region (300–540 amino acid residues), resulting in the recruitment of IKKε and the subsequent phosphorylation of IRF3 and that laboratory of genetics and physiology 2 competes with IKKε to interact with MAVS at the same region to inhibit antiviral RLR pathways [82]. Interestingly, IKKε has been previously reported to colocalize strongly with MAVS on mitochondria upon RNA virus infection, which was disrupted by the hepatitis C virus protein NS3-4A [18]. This was not observed with TBK1 in virus infection, indicating that it rather has an indirect association with MAVS mediated by other adaptor proteins, such as STING or tumour necrosis factor receptor-associated factor 3 [18,73, 74, 83]. A follow-up study revealed that, upon virus infection, MAVS is subjected to K63-linked ubiquitination of the 500-residue site, leading to the mitochondrial recruitment of IKKε, where it interacts with MAVS through its C-terminus, causing its phosphorylation. This has been found to reduce the expression of ISGs, IFN-β and, surprisingly, IL-6, which suggests negative regulation of the NF-κB pathways [83]. Nevertheless, MC089 could use the same principle to direct IKKε to MAVS, either through interaction or via TBKBP1-NAP1, resulting in MAVS phosphorylation and suppression of IRF3 activation, a potential avenue for further study.

Although the precise mechanism of MC089 inhibition through interaction with IKKε, TBKBP1, NAP1 and MAVS is unclear, we found that MC089 inhibits IRF3 phosphorylation at serine 396 without affecting the phosphorylation of serine 386. Such specific targeting could indicate differences in the functional specificity of these two sites. Even though both Ser386 and Ser396 are considered critical for IRF3 dimerization and phosphorylation, the exact role of each site is still debated. It has been reported that IRF3 Ser396 causes IRF3 oligomerization, leading to a strong interaction with the coactivator CREB-binding protein (CBP)/p300, while pIRF3 Ser386 does not associate strongly with (CBP)/p300 but rather strengthens pIRF3 Ser396 binding to the coactivator [40]. In contrast, a study using phosphorylated mutants of the two sites demonstrated that, while both sites are important for IRF3 dimerization, Ser386 is necessary for IRF3 activation [39].

Even though TBK1 phosphorylates IRF3 Ser396 [23,24, 28, 84], MC089 specifically targeting IKKε and pIRF3 Ser396 may indicate that this kinase can also phosphorylate this site, potentially in the context of MAVS-associated activation. Several viral inhibitors target IKKε and MAVS, resulting in inhibition of pIRF3 Ser396. For example, Epstein–Barr virus protein BFRF1 binds IKKε and inhibits pIRF3 Ser396 and SeV-induced IFNβ expression [19]. The Middle East respiratory syndrome coronavirus accessory protein ORF8b inhibits pIRF3 Ser396 and IFNβ production via inhibition of heat shock protein 70 required for IKKε activation [20]. Rotavirus non-structural protein 1 (RNP1) degrades MAVS through direct interaction and inhibits pIRF3 Ser396 and IFNβ promoter activation [22]. IKKε- and MAVS-linked pIRF3 Ser396 have been also demonstrated through the scaffold protein FAS-associated factor 1 (FAF1), which acts as a negative regulator of MAVS by blocking its ubiquitination through the ligase TRIM31. Upon virus infection, IKKε causes FAF1 degradation by direct phosphorylation at serine 556, resulting in its release from MAVS. Interestingly, mutations of Ser556 prevent IKKε-dependent FAF1 degradation, which blocks efficient TBK1 and IRF3 Ser396 phosphorylation [85]. MC089 may use a similar inhibitory mechanism by mimicking FAF1 to negatively regulate MAVS. Alternatively, MC089 interaction with IKKε could prevent the kinase from degrading FAF1.

In summary, the MCV-derived protein MC089 is a novel inhibitor of IRF3 activation by association with MAVS, IKKε and its scaffold proteins TBKBP1 and NAP1. Further investigation into its precise mechanism of inhibition may offer novel insights into IRF3 regulation; in particular, the roles of the constituents of its activating complexes in different nucleic acid-sensing pathways and its model of inhibition may offer a basis for potential therapeutics targeting selective routes to IRF3 activation in diseases and disorders.

## supplementary material

10.1099/jgv.0.002015 Uncited Fig. S3.

## Abbreviations

AP-MS: affinity purification and mass spectrometry
CBP: CREB-binding protein
cGAS: cyclic GMP–AMP synthase
DMEM: Dulbecco’s Modified Eagle Medium
dsDNA: double-stranded DNA
ER: endoplasmic reticulum
FAF1: FAS-associated factor
FDR: false discovery rate
IKK: IκB kinase
IKKε: I𝜅B kinase epsilon
IP: immunoprecipitation
IRF: interferon regulatory factor
ISG: IFN interferon-stimulated gene
ISRE: interferon-stimulated response element
LAM: luciferase assay mixture
LFQ: label-free quantification
MAVS: mitochondrial antiviral-signalling protein
MCV: molluscum contagiosum virus
NAP1: NF-kappa-B-activating kinase-associated protein 1
NEMO: NF-kappa-B essential modulator
PRRs: pattern recognition receptors
RIG-I: retinoic acid-inducible gene I
RLRs: RIG-I-like receptors
SEAP: secreted embryonic alkaline phosphatase
SeV: Sendai virus
SINTBAD: similar to NAP1 TBK1 adaptor
STING: stimulator of interferon genes
TANK: TRAF family member-associated NF-kappa-B activator
TBK1: tank-binding kinase 1
TBKBP1: TBK binding protein 1
TI-IFNs: type I Interferons
TLR3: toll-like receptor 3
TLRs: toll-like receptors
TRAF: TNF receptor-associated factor
TRIF: TIR domain-containing adapter-inducing IFNβ
VVC6: Vaccinia virus IRF activation inhibitor C6
