# Supplementary material for: Molluscum contagiosum virus protein MC089 inhibits interferon regulatory factor 3 activation
Source: J Gen Virol. 2024 Aug 21;105(8):002015. doi: 10.1099/jgv.0.002015 (PMC11338640; doi:10.1099/jgv.0.002015)
Supplement: Uncited Fig. S3. [file jgv-105-02015-s001.pdf]

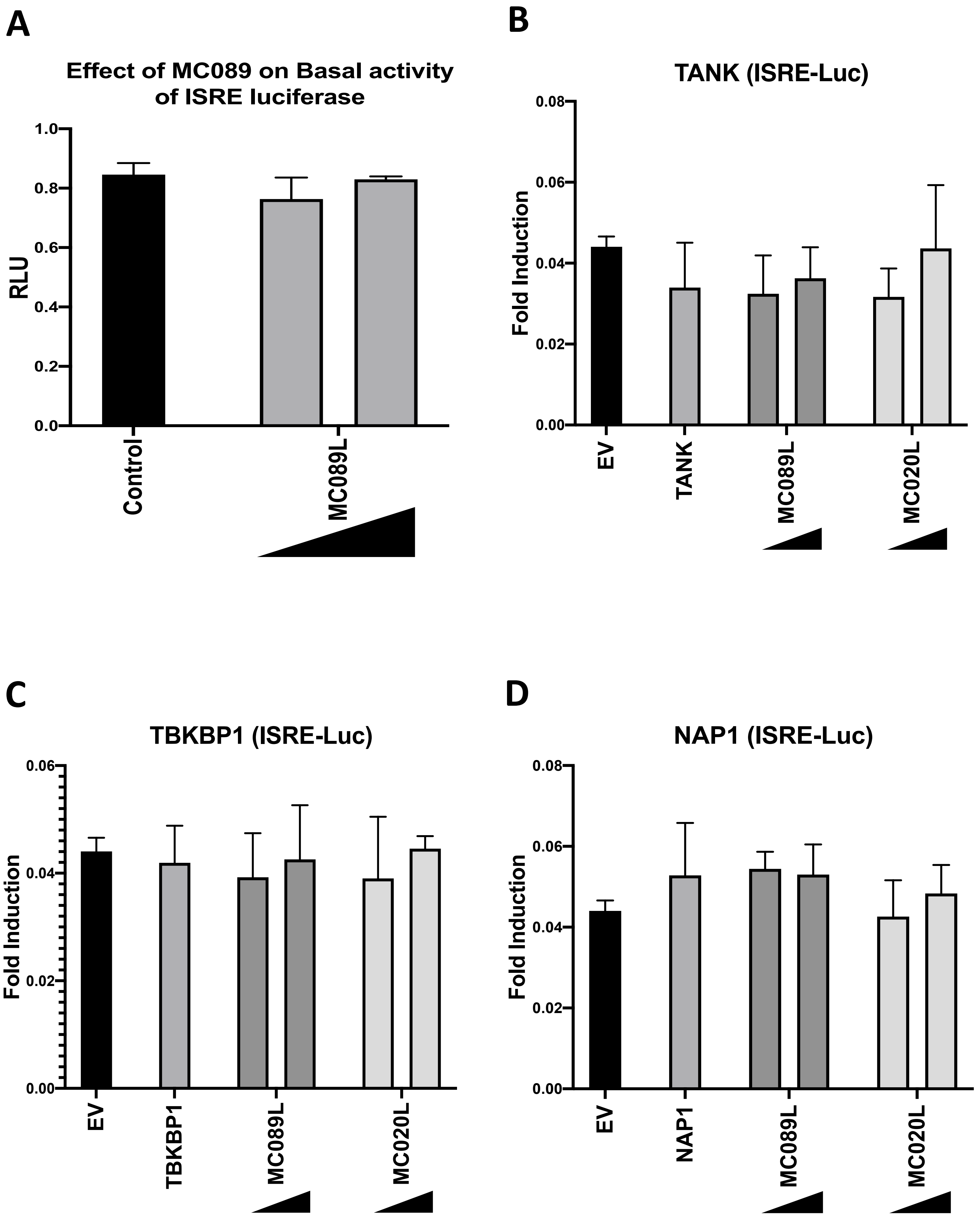
A
Effect of MC089 on Basal activity of ISRE luciferase
RLU
1.0
0.8
0.6
0.4
0.2
0.0
Control
MC089L
B
TANK (ISRE-Luc)
Fold Induction
0.08
0.06
0.04
0.02
0.00
EV
TANK
MC089L
MC020L
C
TBKBP1 (ISRE-Luc)
Fold Induction
0.06
0.04
0.02
0.00
EV
TBKBP1
MC089L
MC020L
D
NAP1 (ISRE-Luc)
Fold Induction
0.08
0.06
0.04
0.02
0.00
EV
NAP1
MC089L
MC020L

Figure S1. Effect of MC089 on basal activity and TANK/ TBKBP1/NAP1 stimulation of ISRE luciferase activation. (A-D) HEK293T cells were seeded at $2x10^5$ cells /ml and transfected 24 hours later with 80 ng of ISRE luciferase reporter. To normalize firefly luciferase, 40 ng of pGL3-*Renilla* control was utilized. Cells were transfected with two doses of pCEP4 constructs expressing MC089 or MC020: 25 ng and 50 ng. (B-D) TANK, TBKBP1 and NAP1 plasmids were added accordingly: (B) TANK (50 ng), (C) TBKBP1 (50 ng) and (D) NAP1 (50 ng). The empty vector (indicated by EV) was used as a control of pathway activation. The total amount of DNA was adjusted to a final volume of 220 ng using the empty vector control (pCMV-HA). Cell lysates were harvested and assayed for ISRE luciferase activity. Schematics are representative of three or more individual experiments. Firefly luciferase activity was normalized to *Renilla* luciferase activity. Bars indicate mean ± the standard deviation. (A) Data are presented by relative light unit (RLU). (B-D) Data are presented by fold induction.

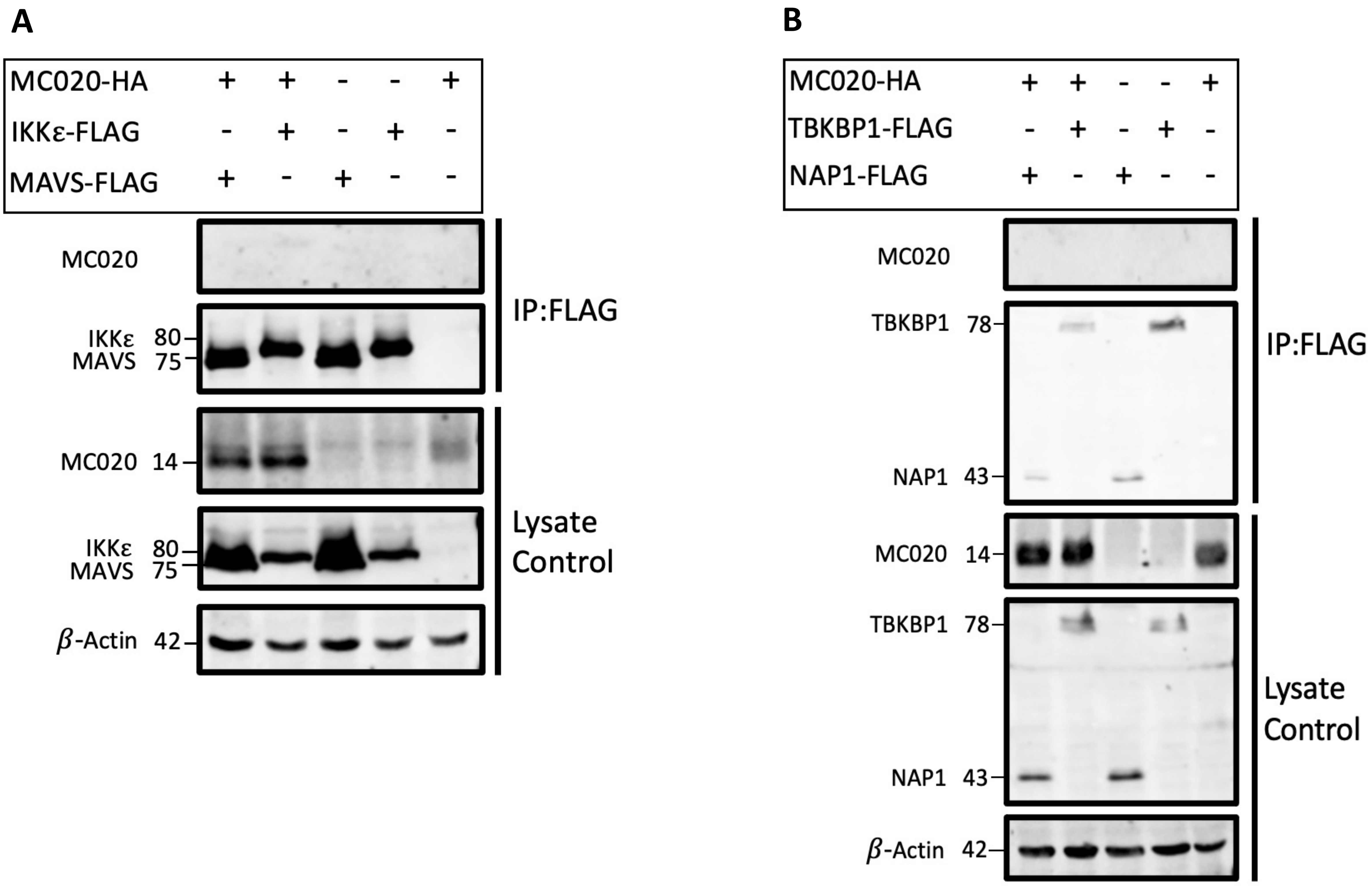
A
MC020-HA + + - - +
IKKε-FLAG - + - + -
MAVS-FLAG + - + - -
MC020
IKKε 80
MAVS 75
IP:FLAG
MC020 14
IKKε 80
MAVS 75
Lysate Control
β-Actin 42
B
MC020-HA + + - - +
TBKBP1-FLAG - + - + -
NAP1-FLAG + - + - -
MC020
TBKBP1 78
NAP1 43
IP:FLAG
MC020 14
TBKBP1 78
NAP1 43
Lysate Control
β-Actin 42

Figure S2. MC020 does not interact with IKKε, MAVS, TBKBP1 and NAP1. (A & B) HEK293T cells were seeded at $3x10^6$ cells/culture dish and transiently transfected 24 hours later with a total of 8 μg of pCEP4-MC020-HA and the indicated signalling pathway component FLAG-tagged plasmids. After 24 hours, cell lysates were immunoprecipitated using anti-FLAG M2 affinity gel beads, eluted with FLAG tag peptide, and probed with the appropriate antibodies: anti-HA (first and third panels), anti-FLAG (second and fourth panels) and anti-β-actin (fifth panel).
